# Uniform Veterinary Education*Read before the Keystone Veterinary Medical Association, April 4, 1891.

**Published:** 1891-05

**Authors:** W. Horace Hoskins


THE JOURNAL

OF

COMPARATIVE MEDICINE AND VETERINARY ARCHIVES.

VOL. XII. MAY, 1891. NO. 5.

### UNIFORM VETERINARY EDUCATION.*

By W. HORACE HOSKINS, D. V. S.

When I received the notice of this meeting from our worthy temporary secretary, conveying to me the information that I was to enlighten you this evening on the topic assigned for consideration at this time, I was as much astonished as I was forcibly impressed of my inability to afford you an evening's thought on so abstruse, so needful and so important a subject. With all these hurried thoughts rushing through my mind, it seemed to me an intangible mass, from which to work out some plan that was feasible in this wonderful country of ours. First came in my mind the legality of such a movement and whether such a National Board could be in existence with any power that would still work grander the veterinary structure we are rearing in America. Then from what source should this power come? The tendencies of recent years for a more centralized government in this country, has almost already awakened minds to rebellion, and every step in this direction is seized upon to-day by those of democratic ideas of government, to mark the faltering strength of our republican form of government, and the moving of authority away from the masses, pointed to as a lack of faith in the government under which we have grown to such a remarkable place in the world's history that, our career has dazzled the eyes of the potentates of

*Read before the Keystone Veterinary Medical Association, April 4, 1891.

every nation under the sun. By what power this body would be perpetuated and kept marking out a higher and broader sphere, and saved from deteriorating forces, that so often have followed in the history of similar organizations, or to be freed from the ensnaring and compromising influence of our spoils system, both by those who destroy by fawning, as well as by those more to be respected enemies, who fight openly and persistently the system under which we would hope only to work, that of merit and worth, all kept crowding one another to make more obscure the impenetrable maze that surround this subject. I had hoped the efforts put forth to have before you to-night, one of the foremost enthusiasts in the advancement of veterinary science in our country, had been successful and I would have had the pleasure of listening to the unfolding of his plans, for our future betterment and the strengthening of our position in the world of science.

This left one very ardent hope in my mind and I thought it commanded me in offering you my views on this subject to-night; it was the hope that among you, the Keystone members, some one would reach up to the full measurement of a plan, that would accomplish our hopes and wishes as veterinarians and we might as fellow-members indulge in the pleasure of sitting in his reflected glory and, one more laurel might be added to the honor of our association.

This subject of a single standard of education is not a new one in this country. As early as 1876 we find a number of the leading men, in the ranks of scientific workers for our profession, and filling valuable places in the channels of instruction for those who were to become a part of the veterinary profession, looking with a single eye to the good of our calling, sitting in deliberations over a single plan to achieve the acme of this work. At that time in North America the task seemed easier than now. A fewer number of schools existed and it only seemed necessary then for a single move on the part of three or four leaders, to consent to a lengthened course of study that would have answered the problem for future years. But it was a failure, and the intervening period from 1876 to almost 1886 it slumbered the sleep, that knows no waking and left for a future generation the solving of this knotty problem.

In 1884, as Chairman of the Committee on Intelligence and Education, in a report read at the September meeting in Cincinnatti, Ohio, I suggested the wisdom of the schools of North America teaching veterinary science, to meet in conference, for the purpose of considering a single standard of requirements. From this report grew the now fixed committee of that association which for seven years has labored to bring together representatives of these various schools, but all to no avail. I was honored for some three years with the chairmanship of that committee and my youth in the profession, coupled with a loving enthusiasm for my chosen avocation, required some three years of continued failure to chill my ardor in this much needed work. Others have followed with equal zeal only to learn as I had done the stumbling blocks to success. But I shall not forget here to place on record the aid and sympathy, full of honest acquiescence in any move that would accomplish the desired end, offered by several of the heads of leading veterinary institutions in North America. Nor shall I hesitate to say that those who proved the obstacles then are the ones who stand to-day in the way of the accomplishment of this work, and yea more; others are sowing the same seed, that they were scattering then, and justify themselves in their work, by the example of those who were earlier in the fields, seeking numbers, not few; wanting quantity, not quality; desiring sordid gain, rather than the hope of a place in posterity. Now the scenes have shifted and, it becomes our duty to endeavor to lift up the profession to a higher standard of requirements, than the sources from which many of us have emanated.

One year of work on that committee led me to see the hopelessness of a single standard of requirements, for preparation for the responsibilities of our work, and I then gave the remaining two years to the work of achieving at least a similar grade of entrance examinations and a minimum course of time for college work; but when confronted with the views of one of the leading institutions of veterinary science as expressed by its chief: "that he would rather have blacksmiths as students, than any others; as they made the best veterinary surgeons," you can form some idea of the hoplessness of my task and with what pleasure I laid down my duties for stronger hands, I hope, to carry to a more successful termination.

This brings me to the point of our subject for to-night, for I have lost faith, though reluctantly, in the forces that to-day control the veterinary schools of North America, and can see no floating beacons on the current of veterinary science as it surges through our entire country to-day, of a hope of any united effort on the part of the colleges themselves, for a unity of purpose in work and achievements; for a grander, nobler, broader, stronger profession; fitted for the grave responsibilities of to-day and the graver ones before us in the future of to-morrow. But I do see a growing tendency in the development of mush-room schools in all sections and even in States, that forebode much evil and promise only to dwarf the progress of American veterinary science in every branch and channel of its work. I see its tendencies leavening the whole mass that make up our sources of instruction; the alluring baits held out for material to make the veterinarians of the future, make uncertain, unsafe the position of the schools in existence to-day and by lessening their sources of income, not profit, they check the disposition to broaden the curriculum, or lengthen the course of study, lest it deter students from entering and affording them a ready excuse. I have often heard expressed that they selected the easiest schools, because of the greater assurance of their being able to get through, and becoming a full-fledged doctor; little realizing the greater responsibilities before them in the field of labor. Why, gentlemen, the courses of some schools to-day are not long enough, nor broad enough to rise up to the full needs of preparation for the work, aside from the consideration of the time of a completed education. This leads us to the point of the duties of such a Board. I may be pardoned here in quoting the remarks of the Chief of our Bureau of Animal Industry, made in Chicago, in 1890, at the Convention of the U. S., V. M. A., "that should the bill then pending before Congress, outlining a plan for examining our food supply, principally our meat and milk, that he did not know where he could find men enough to do a tithe of the work," and I would add, from where in the ranks of the average American veterinary graduate could he find men competent to do the work? I shudder at the thought of the responsibilities that are soon to be heaped upon us as a profession, lest the work we were intended to do, should become a farce in the eyes of the scientific world and our profession the butt of ridicule or reproach. A National Board of Examiners to-day would only complete its work for our people, when it would send forth with its insignia, a man drilled as a veterinarian in the every-day work of our calling; trained as a surgeon in the application of mechanical skill for the relief he was destined to give; schooled in the knowledge of medicine and physics the domain of the volume of his work; fitted by experimental training in exercising the duties of a sanitarian; whether in the confines of a building for shelter, the field of hidden sources of danger for our food supply and the place of preparation for consumption of those products which largely mould the strength and power of a Nation.

Is it not time for a veterinarian entering our ranks to-day to be so equipped, and where in our Nation to-day will he turn for such an education? In one centre he can secure a good preliminary education; in another he can learn well to treat the diseases of the equine species; in another he can secure a good training in surgery; in another he can secure a solid education in all the adjunct branches of our science; while in several others he can secure a smattering of all these, almost wholly from a series of lectures only. What would you think of a carpenter coming to your house to-day, to hang a door, who had never placed one in his career as an apprentice; what would you think of a plumber who would come to your home to place a drain, who had never laid a pipe in his days of being fitted for his work. Yet I can point to you to-day hosts of veterinarians who have emanated from their college parents armed with sheepskins, who never performed a single foot operation in their college career, whose eyes never covered the field of a microscopic slide, whose hands never compounded a ball of physic or placed a bandage on a limb, for even the purpose of warmth of sustaining peripheral circulation. Are these not sufficient reasons for a higher body to-day that shall give assurance to our people that we have qualified veterinarians, and afford to those who contemplate a place in our ranks for the good of the work he may do, a higher goal to aim for and a more valuable franchise than the average sheepskin that confers upon us the title of Doctor. Stepping aside for a moment, we may consider the value it may ultimately be in the creation of a single title and degree; thus casting aside the confusing degrees of D. V. M., B. V. M., V. M. D., D. V. S., B. V. S., V. S., and other conflicting titles that mar the uniform claim we all possess as veterinarians and lead among the laity, the disposition to place a relative value on the number of letters significant of our title, as well as to the complex way in which they are placed after our names; rather than to a consideration of the value of the sources from which we received our education.

Now shall this National Board be vested with authority by our government, by an act, finding its authority for creation at the hands of Congress? How far could such a body go, before trespassing upon State rights, so long as our schools are created solely by State jurisdiction? Would its creation lead to a supervision of all schools by the general government and ultimately grants of powers and monies to make them complete. Could this be achieved under a republican form of government? Many will answer no! Many will answer yes, and point in support of the same the tendencies toward general laws for food inspection and adultration; the agitation for government supervision and perhaps ownership of our telegraph system, and rising before us with such astonishing magnitude a new party, looking forward to the supervision of our railroads by the general government and asking you why not? when fully as many people in these rapid times use the railroads as use the post-office system, both of which are essential for our progress and welfare and equally useful. Personally I do not look with favor on its entire creation in this way, but would rather favor its chief being selected in this way only and government power being vested in it to the fullest extent permissible, with all deference to State rights; this power being rather in the direction of usefulness in fixing the standard of requirements and its perpetuation. This has one merit of making the source of its sustenance an easy problem, by placing it upon the National Treasury, but in these days I fear it would be an endless time in the distance before it could be achieved. Again, I have thought the Bureau of Agriculture might find it within the scope of its work to give this a resting place under it broad wings, under the claim of its field being so broad as to not only include in its domains, the health and longevity of our people; but the importance and value commercially of our live-stock interests. While this would answer for its creation I can hardly realize yet how it would bring to its feet the work it was destined to accomplish.

Then again, I have conceived of a government chief, with State Boards as adjuncts; these to be based upon the general plan as in effect to-day for the completion of a true Civil Service. The standards of requirements being fixed by a government head, and say two assistants, and then the work being put in application by the State Boards. This would involve the necessity of States accepting the provisions of the National law, passing laws for the creation of a State Board, these State Boards to be paid as State officers and to consist of three members; one to be appointed by the college or colleges jointly or in their not being any, by the State Association; one by the Governor and the third selected by these two. This plan would have the merit of least interference of State's rights and would only have as a grave objection the expense it would entail upon the State. Though this would in some measure be offset by, a fee being exacted before permission is granted for examination. These fees not to paid to the Board, but to the General Treasury; lest their number might be a temptation to the Board and bring forth the objections raised to-day to our Board of Pharmacy act; and the fees being exacted by County officers, that led to such indiscriminate registrations in the rural districts under the provisions of our State Veterinary Act.

Last of all, and that which has appealed most strongly to my feelings and belief in the sovereignty of the people, has been the creation of such a body, through the uplifting of our voices as veterinarians throughout the entire land, for its creation. Arising from the popular will of our own members, no school will long dare to defy its commands, nor turn itself away from it with scorn. Coming as it would from the outgrowth of a realization of its needs by the rank and file of the profession it would be destined to live a longer career when upheld and sustained by such a support. I would clothe such a Board with power to define who should become members of our United States Veterinary Organization. I should make it necessary by government statute for all applicants for positions in the army service, to be clothed first with its insignia; before being privileged to become competitors for a place, the same in our Bureau of Animal Industry, and thus remove it from the bane to-day of our infamous spoils system, and for positions on Sanitary and State Boards of Health, for Milk and Meat Inspectors and for positions as State Veterinarians. Its source of perpetuation might well emanate from the National Association of Veterinarians, thus giving it a fixed means of continuance. Our country might be then divided into a Central and an Eastern and Western division. To these might be delegated limited authority under such provisions as might prove useful to conduct the examinations for the central body. To have their graduates prepared so as to be able to pass this Board would in time be the goal and aim of all veterinary colleges and its passport the only one of worth in our country.

Make it the commanding necessity of every man selecting the veterinary profession as a calling, to use such discretion in his selection of a school, that would fit him for this higher examination; that some schools in existence to-day would have to take a broad step forward or drop out of the fight, and schools for private gain, self-vanity, and gratification, would be things of the past. Its authority emanating solely from the people, would prove the strongest in the end and sustained by a united profession it would soon solve the question of a higher education for the future members of our calling, and its commanding position lift us up to higher altitudes than we have yet touched. Its number might be fixed at seven, with three for the profession outside of schools and four for specialists engaged in teaching, we could be assured of a fair hearing and just decision.

When you contemplate that four or more years are to-day exacted in learning the trades and many of the other avocations, when certain medical schools are considering the advisability of a four year's course, when the scope of knowledge to be had to-day in any branch of science has so broadened and deepened, when the old system once in vogue of apprenticeship in our profession has fallen into disuse, are you not convinced, that two short years' of five and a half, to seven month's, or a three years' course of five month's, are far too short for the education of a man to the ranks of a profession with the responsibilities of the magnitude that are ours to-day? Is it not time for every organization in our land banded together for the advancement of our cause, to rise up to the full dignity of its labors and demand the creation of some single higher body than to-day exists for the settlement of this question of a higher education? If colleges will not lift themselves up, if they have no greater devotion for the profession, than some are displaying; then it becomes no less our duty to adopt such means as will achieve this end.

